# KW-2449 and VPA exert therapeutic effects on human neurons and cerebral organoids derived from MECP2-null hESCs

**DOI:** 10.1186/s13287-022-03216-0

**Published:** 2022-12-27

**Authors:** Ya-Jie Xu, Pei-Pei Liu, Zhong-Ze Yan, Ting-Wei Mi, Ying-Ying Wang, Qian Li, Zhao-Qian Teng, Chang-Mei Liu

**Affiliations:** 1 State Key Laboratory of Stem Cell and Reproductive Biology, Institute of Zoology, Chinese Academy of Sciences, Beijing, 100101 China; 2 Savaid Medical School, University of Chinese Academy of Sciences, Beijing, 100049 China; 3 Institute for Stem Cell and Regeneration, Chinese Academy of Sciences, Beijing, 100101 China; 4 Beijing Institute for Stem Cell and Regenerative Medicine, Beijing, 100101 China

**Keywords:** MECP2, Rett syndrome, Neural differentiation, Cerebral organoids

## Abstract

**Background:**

Rett syndrome (RTT), mainly caused by mutations in methyl-CpG binding protein 2 (MECP2), is one of the most prevalent neurodevelopmental disorders in girls. However, the underlying mechanism of MECP2 remains largely unknown and currently there is no effective treatment available for RTT.

**Methods:**

We generated MECP2-KO human embryonic stem cells (hESCs), and differentiated them into neurons and cerebral organoids to investigate phenotypes of MECP2 loss-of-function, potential therapeutic agents, and the underlying mechanism by transcriptome sequencing.

**Results:**

We found that MECP2 deletion caused reduced number of hESCs-derived neurons and simplified dendritic morphology. Moreover, MECP2-KO cortical organoids exhibited fewer neural progenitor cells and neurons at day 60. Electrophysiological recordings showed that MECP2 deletion altered synaptic activity in organoids. Transcriptome analysis of organoids identified many genes in the PI3K-AKT pathway downregulated following MECP2 deletion. Treatment with either KW-2449 or VPA, small molecules for the activation of PI3K-AKT signaling pathway, alleviated neuronal deficits and transcriptome changes in MECP2-KO human neuronal models.

**Conclusions:**

These findings suggest that KW-2449 and VPA might be promising drugs for RTT treatment.

**Supplementary Information:**

The online version contains supplementary material available at 10.1186/s13287-022-03216-0.

## Introduction

Rett syndrome (RTT) is a severe progressive X-linked neurodevelopmental disorder with mental retardation in girls, which is characterized by a period of early normal development followed by a regression phase, leading to loss of speech, impaired motor function and purposeful hand movements, intellectual disability, seizures and microcephaly. In the majority of 90–95% cases, it is caused by loss of function of the methyl-CpG binding protein 2 (MECP2) [1, 2].

MECP2 plays essential roles in transcriptional regulation and chromatin remodeling, alternative splicing, and microRNA processing [3–5]. Although MECP2 is expressed in all cell types, the lack of neuronal MECP2 is thought to account for the majority of symptoms associated with RTT [6, 7]. In RTT, BDNF signaling is impaired, and BDNF and its regulators (such as histone deacetylases HDACs) are speculated as therapeutic targets for RTT syndrome [8]. Indeed, BDNF is critical for neuronal development, maturation, and plasticity through the activation of TrkB, which in turn activates PI3K/Akt pathways that regulate protein synthesis and neural function [9]. However, BDNF cannot cross the blood brain barrier, thus there is no immediate therapeutic application for RTT patients [10]. Given that therapies aiming to restore MECP2 loss-of-function are still at an early development stage, there is a great need to develop human neuronal models for accurate and comprehensive mapping of the MECP2 network which can be used for screening interventions [8, 11].

Using 2D-based neuronal differentiation and 3D cerebral organoid culture platforms certainly contribute to a better understanding of the pathogenesis and the disease phenotype of RTT, ultimately allowing the development of drug tests for potential clinical translation [12]. Here we generated human neuronal models of RTT using a combination tool of genome-edited human embryonic stem cells (hESCs) and subsequent neural differentiation. We demonstrated that the downregulated PI3K-Akt pathway is responsible for impaired neuronal differentiation and cell morphology in human neuronal models upon MECP2 deletion. Importantly, application of KW-2449 or VPA, small molecules for the activation of PI3K-AKT pathway, could alleviate neuronal deficits in MECP2-KO human neuronal models.

## Materials and methods

### Cell cultures and drug treatment

The H9 ESC line (WA09) was a gift from Prof. Baoyang Hu at the Institute of Zoology, Chinese Academy of Sciences. Cells were cultured on Matrigel (BD) coated with TeSR™-E8™ media (STEMCELL Technologies, 05990). Cells were passaged every 4 days by EDTA (100 mM) treatment.

For drug treatment of 2D neuronal cultures, 1 μM KW-2449 (Selleck, S2158) or 10 μM VPA (MedChemExpress, HY-10585) was added to the medium starting from day 20 of neuronal differentiation and medium was replenished every two days. Neurons were collected at day 40. For drug treatment of brain organoids, 1 µM KW-2449 or 10 μM VPA was added to the medium starting from day 11, and drug-containing medium was replenished every four days.

### Neuron differentiation

Neural differentiation was performed as described previously with minor modifications [13]. Briefly, the hESCs were dissociated as single cells in hESC medium with 10 µM Rock inhibitor, Y27632 (Selleck, S1049) to form embryoid bodies [14]. At day 2 to 7, the hESCs medium was replaced with N2 medium (DMEMF/12, Gibco, 10565042; 1xN2, Gibco, 17502048; 1xNEAA, Thermo Fisher, 11140-050). Then the EBs were plated at day 7 on Matrigel-coated plates with 1 µg/mL laminin (Gibco, 23017015) in N2 medium. Neural progenitor cells (hNPs) in the form of rosettes were picked by collagenase IV (Gibco, 17104019) at day 14 and expanded as neurospheres in N2B27 medium containing DMEM/F12, 1xN2, 1xB27 (Gibco, 17504044), 0.2 µM RA (Sigma, R2625) and 20 ng/mL FGF2 (Peprotech, AF-100-18B-100) until day 20. For neuronal differentiation, the dissociated hNPs were plated onto poly-L-ornithine/laminin (50 µg/mL)-coated coverslips with neuronal differentiation medium (DMEM/F12, 1xN2, 1xB27, 10 ng/mL BDNF, 10 ng/mL GDNF, 1 mM dibutryrl-cyclic AMP and 200 nM ascorbic acid) (Additional file 1: Fig. S1A).

### Brain organoid culture

Organoid culture was used STEMdiff Cerebral Organoid Kit (STEMCELL Technologies, 08570) according to the manual. Briefly, hESCs were dissociated into single-cell suspension using accutase, and then 9000 cells were plated into each well of 96-well plate for EBs formation. At day 7, EBs were embedded in Matrigel and cultured in the expansion medium for 3 days and then in maturation medium which was renewed every 4 days.

### MECP2 knock-out in hESCs

We designed two sgRNAs to targeting *MECP2* using the website http://crispr.mit.edu [15]. Two sgRNAs coding sequences were respectively cloned into the PX330-GFP-U6 plasmid obtained from Professor Haoyi Wang at the Institute of Zoology, Chinese Academy of Sciences. Two million of single hESCs were electroporated with 7 µg of PX330-sgRNA using the Human Stem Cell Nucleofector® kit 2 (Lonza) with program CM115 in a Nucleofector II device. After electroporation, cells were plated onto Matrigel-coated plates with hESCs medium containing 10 μM Y27632. GFP-positive cells were selected with Flow Cytometry and reseeded as single cells starting from 36 h after electroporation. Single clones were picked and expanded for PCR amplification of target region.

### Immunostaining

Organoids were collected, post-fixed in 4% paraformaldehyde (PFA, in 0.1 M phosphate buffer, pH 7.4) at 4 °C for 12 h, and then dehydrated in 30% sucrose in PBS at 4 °C. Organoids were cut into 15 µm sections mounting on freezing microtome (Leica CM 1950). For immunostaining, the organoid slices were washed in PBS, permeabilized with 0.5% Triton X-100 for 15 min and blocked in a buffer containing 3% bull serum albumin and 0.3% Triton X-100 for 1 h, and incubated with primary antibodies at 4 °C overnight. The brain slices were washed with PBS and incubated with Alexa Fluor-conjugated secondary antibodies for 1.5 h at room temperature. The sections were washed with PBS three times and mounted in adhesion anti-fade medium. For immunostaining of 2D cultures, coverslips were washed with PBS for three times followed by 4% PFA for 15 min. After washing for three times, coverslips were blocked in blocking buffer containing 3% bull serum albumin and 0.3% Triton X-100 for 1 h, and then incubated with primary antibodies at 4 °C overnight. The coverslips washed by PBS and labeled with the secondary antibodies for 1.5 h, and finally washed with PBS and then mounted in adhesion anti-fade medium. The immune-stained cells were viewed under a Zeiss LSM 880 confocal imaging system (Zeiss, Germany) at an objective magnification of 20 × . ImageJ software was used to calculate the positive cells.

### BrdU administration

To examine the proliferation ability of hESCs and hNPs (at day 14 of neural differentiation), hESCs and hNPs were trypsinzed into single cells, and plated as monolayers on glass coverslips coated with Matrigel. 5 µM BrdU (Sigma-Aldrich, St. Louis, MO, USA) was added into the culture medium for 3.5 h.

### TUNEL assay

The TUNEL assay was performed using the One Step TUNEL Apoptosis Assay Kit (Beyotime, C1090) according to the manufacturer’s instruction. Briefly, organoid sections were fixed with 4% PFA and permeabilized by TrionX-100. Sections were then incubated with TUNEL reaction mixture for 1 h at 37 °C in dark. Samples were counterstained by DAPI and analyzed under a fluorescence microscope.

### Electrophysiology

Organoids of PX330 and MECP2-KO at day 120 during differentiation were embedded in 4% low melting point agarose and placed in ice-cold solution containing the following (in mM, pH adjusted to 7.4): 92 NMDG, 1.3 NaH_2_PO_4_, 5 KCl, 0.5 CaCl_2_, 26 NaHCO_3_, 10 MgCl_2_, 5 Na-ascorbate, 2 thiourea and 25 D-glucose. Organoids slices (200 µm) were sectioned with a vibratome slicer (Campden instruments, 7000 smz) and were let recover for 30 min at room temperature before recordings. During recordings, slices were bathed in an external solution containing the following (in mM): 124 NaCl, 1.5 MgCl_2_, 3.3 KCl, 26 NaHCO_3_, 1.3 NaH_2_PO_4_, 11 glucose and 2.5 CaCl_2_, with pH 7.4. The external solution was bubbled continuously with 95% O_2_ and 5% CO_2_. The patch pipettes (resistances of 5–6 MΩ) were filled with an intracellular solution containing the following (in mM, pH 7.4): 115 cesium methanesulfonate, 15 CsCl, 2 MgCl_2_, 10 HEPES, 10 EGTA, 4 ATP-Mg and 1 QX-314. Cells were voltage-clamped at − 60 mV during mEPSC recordings. Organoids were recorded using a Multi-clamp 700B amplifier and an Axon Digidata 1440 A digitizer. Bicuculline (10 μM), tetrodotoxin (TTX, 1 μM) and APV (2amino-5-phosphonovalerate, 50 μM) were added to the extracellular solution to isolate AMPA-receptor-mediated mEPSCs. All experiments were carried out at room temperature (23 °C). The cell capacitance and series resistance were compensated. The pCLAMP software suite (Version 10.6; Axon Instruments, CA, USA) was used for data acquisition and analysis.

### RNA sequencing and bioinformatics analyses

Total RNAs of organoids were extracted using Trizol reagent according to the manufacturer’s protocol. The quantity and quality of total RNA were assessed using a Nanodrop spectrophotometer. The qualified libraries were loaded on Illumina HiSeq platform for PE150 sequencing. Global transcriptome sequencing was conducted by Annoroad Gene Technology Co., Ltd. FastQC was used to assess sequencing quality control and FASTX-Toolkit was used to remove the reads containing adapter sequences. Alignment-based quantification methods were applied on mappings processed with Salmon (v1.1.0, SAF Pattern). The clean reads were mapped to the reference genome via GENCODE GRCh38 v33. Differential gene expression was performed with DESeq2. Significantly differentially expressed genes were identified with *p*-value < 0.05, fold change > 1.5 or fold change <  − 1.5. Gene function and pathway enrichment analysis were subjected to gene ontology (GO) analysis and Kyoto Encyclopedia of Genes and Genomes (KEGG).

### RNA isolation and qRT-PCR analysis

Total RNA was extracted from collected samples with Trizol (Gibco, 15596018) and cDNA was synthesized using Reverse Transcription Kit (TransScript One-Step gDNA Removal and cDNA synthesis Kit, 20200725) according to manufacturer’s instruction. Real-time qPCR was performed using SYBR Premix Ex Taq (TliRNaseH Plus) and quantitative gene expression analysis used the 2^−ΔΔCT^ method. The mRNA expression levels were normalized to *GAPDH*.

### Western blot analysis

Protein samples were lysed in ice-cold RIPA buffer (ThermoFisher, Waltham, MA, USA) with protease inhibitors (Roche Applied Science). Protein content was determined using a BCA Protein Assay kit (Biomed, PP0102) according to manufacturer’s instructions. Protein samples were separated on 6–15% SDS-PAGE gels and transferred onto PVDF membranes (Millipore). Membranes were blocked in 5% milk in TBS-T (0.05% Tween-20) for 90 min and incubated with primary antibodies overnight at 4 °C. Membranes were then washed three times and probed with secondary HRP antibodies for 2 h at room temperature. Membranes were detected with enhanced chemiluminescence reagent (ECL, Pierce). Protein bands were quantified using ImageJ software. Quantification of protein were normalized to β-Actin or GAPDH.

### Antibodies

The following antibodies were purchased from Cell Signaling Technology: MECP2 (3456 T), PCNA (13110 T). Oct-3/4 (sc-5279) antibody was purchased from Santa Cruz. NANOG (14295-1) antibody was purchased from Proteintech.

### Statistical analysis

The experimentation, quantification, and analysis of data were performed with subjective unbiased methods, and the researchers conducting the experiments were blinded to genotype and treatment conditions except for Western blot analysis. We applied Shapiro–Wilk and Kolmogorov–Smirnov tests to assess normal distributions of datasets. All datasets passed the normality test in the present study. Statistical analysis of the data was generated using GraphPad Prism 8 software. For comparison of multiple groups, one-way analysis of variance (ANOVA) with Bonferroni post hoc correction was performed. The two-tailed Student’s *t*-test was used to determine the statistical significance between different experimental groups. The levels of significance were defined as follows: *p < 0.05, **p < 0.01 and ***p < 0.001. All data are represented as mean ± SEM (standard error of the mean).

## Results

### Loss of MECP2 induces abnormal human neurogenesis in 2D culture

**Fig. 1:**
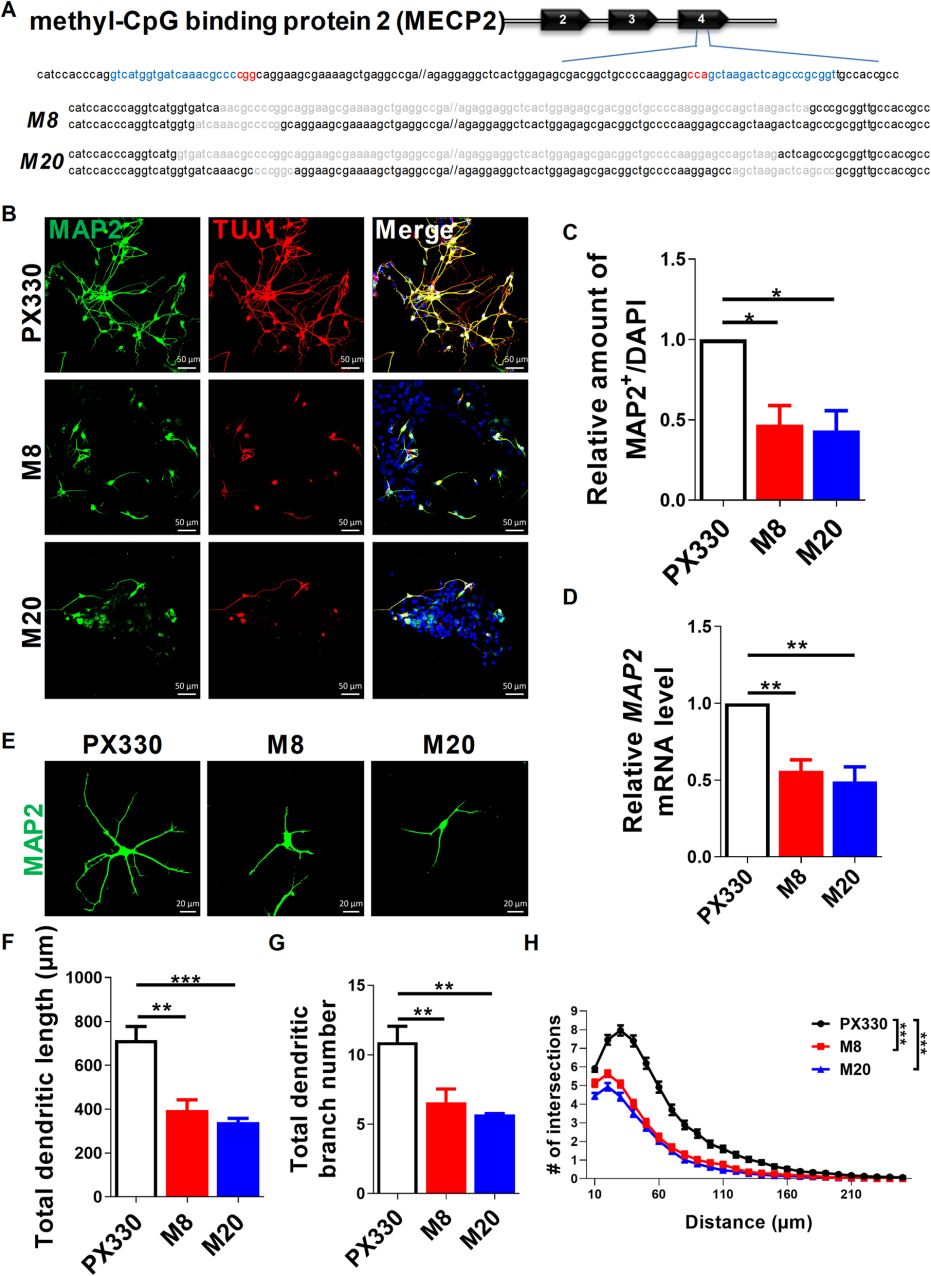
Human neurogenesis is severely impaired upon MECP2 deletion in 2D culture. **A** Schematic view of the strategy to knockout *MECP2* in hESCs using CRISPR/Cas9 (M8, M20). Boxes indicate the exons of *MECP2*, and sgRNA sequences are labeled in blue and the PAM sequences are highlighted in red. Light-colored sequences indicating the deletion of *MECP2* sequence. **B**, **C** Representative images of immunofluorescence staining for neuron markers MAP2 and TUJ1 at day 40 of neural differentiation (B) and subsequent quantitative analysis showing reduced percentages of MAP2^+^ neurons derived from MECP2-KO hESCs (C). *n* = 3 independent experiments. **D** qRT-PCR analysis of the expression of the neural gene *MAP2* in PX330 and MECP2-KO neurons at day 40 of neural differentiation. **E** Representative images of immunofluorescence staining for MAP2 in PX330 and MECP2-KO neurons at day 40 of neural differentiation. **F–H** Quantification of total dendritic length (F), dendrites (G) and dendritic complexity (H) of MAP2^+^ neurons at day 40 of neural differentiation. n > 60 neurons from 3 independent experiments per group. *p < 0.05, **p < 0.01, ***p < 0.001. Data are shown as mean ± SEM; Two-tailed Student’s *t*-test

To explore the functions of MECP2 in human neurogenesis, we firstly conducted CRISPR/Cas9-mediated gene editing to knock out MECP2 in hESCs. Two specific single-gRNA (sgRNAs) targeting exon 4 of MECP2 were designed at Optimized CRISPR Design website (http://crispr.mit.edu) [15]. Western blotting and immunofluorescent staining analyses showed that MECP2 was successfully deleted in two clones of hESCs (named M8 and M20, respectively) (Fig. 1A). Off-target effect was not detected in five potential putative sgRNA genes that were predicted by the Optimized CRISPR Design tool, indicating the specificity of the selected sgRNAs we designed for targeting MECP2 (Additional file 3: Table S1). Compared to the WT, we observed a complete loss of MECP2 protein but a normal karyotype in both M8 and M20 clones (Additional file 1: Fig. S1B–E; Additional file 2: A). No significant differences in expression levels of pluripotency markers between MECP2 KO and WT hESCs were detected by immunofluorescent staining, qRT-PCR and Western blotting (Additional file 1: Fig. S2A–I; Additional file 2: B, C), indicating that MECP2 was dispensable for maintaining pluripotency of hESCs. Bromodeoxyuridine (BrdU) incorporation and pH3S10 immunofluorescent staining analyses illustrated that MECP2 WT and KO hESCs had similar proliferation potentials (Additional file 1: Fig. S3A–D). These results suggested that MECP2 deletion did not affect the pluripotency and self-renewal of hESCs.

To determine the roles of MECP2 in human neuronal differentiation, we induced hESCs to differentiate towards neurons using a previously established protocol [16]. MECP2 WT and KO hESCs were differentiated side-by-side into neural rosettes, and further expanded as neural precursors (hNPs) in the presence of bFGF. hNPs were then cultured for terminal neural differentiation (Additional file 1: Fig. S1A). At day 14 of neural differentiation, both MECP2 WT and KO cells expressed Nestin, an hNP marker (Additional file 1: Fig. S4A). We then checked proliferation of hNPs by BrdU incorporation assay, and found that the proportion of BrdU^+^ hNPs was decreased in MECP2 KO hNPs compared to WT control (Additional file 1: Fig. S4A, B). At day 40 of neural differentiation, we observed a decreased number of microtubule-associated protein 2 (MAP2) positive neurons in MECP2 KO group (Fig. 1B–D), indicating that MECP2 loss-of-function decreased neuronal differentiation efficiency of hNPs. We next determined whether the loss of MECP2 affected neurite growth of hESCs-derived neurons. Morphology analysis showed significant decreases in neurite length and branch number in MAP2^+^ MECP2-KO neurons (Fig. 1E–H).

### Loss of MECP2 impairs neurogenesis in human cortical organoids

**Fig. 2:**
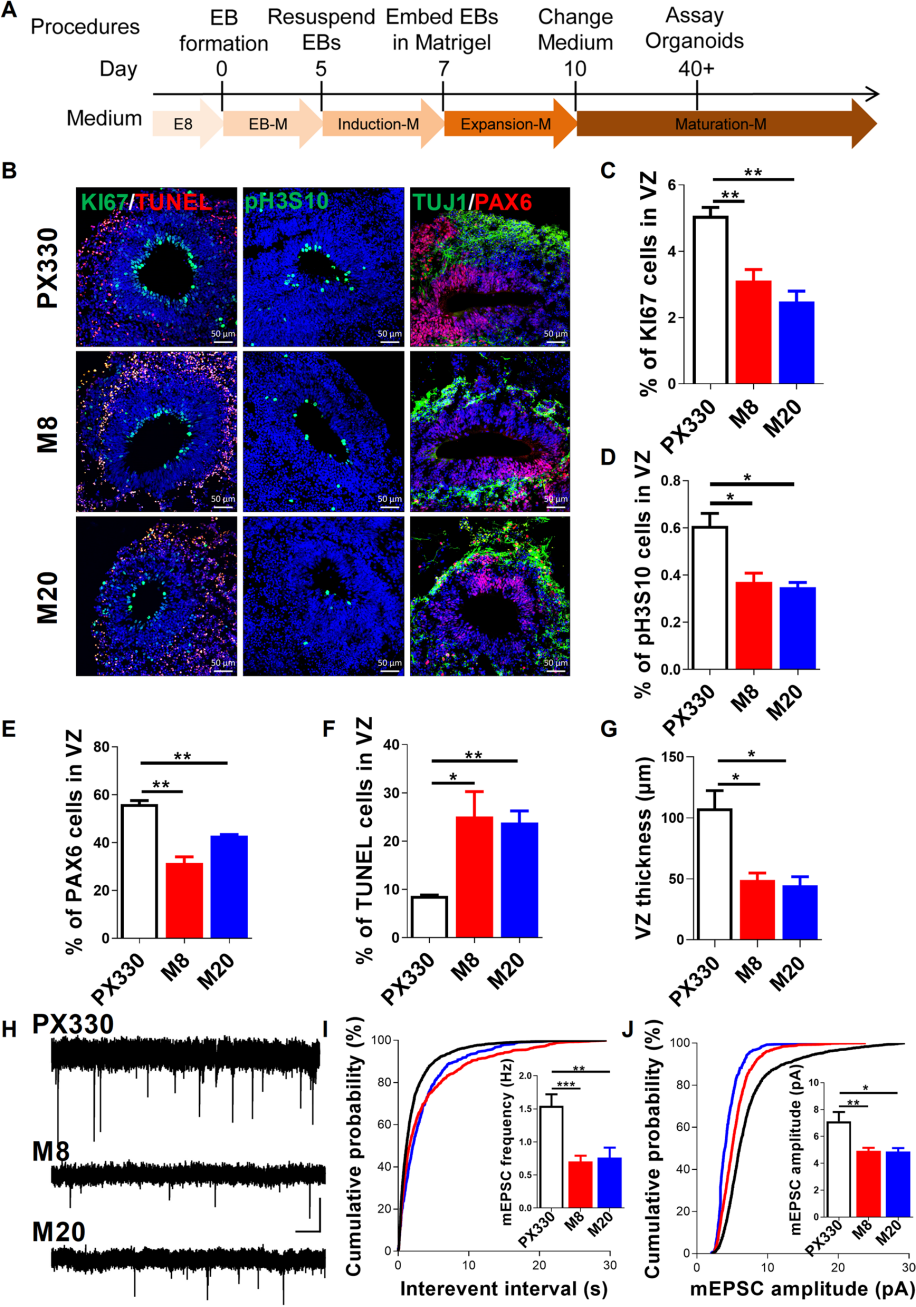
Loss of MECP2 impairs neurogenesis during brain organoid development. **A** Schematic procedure of culturing brain organoids derived from hESCs. **B** Representative images of immunofluorescence staining for KI67, TUNEL, pH3S10, TUJ1 and PAX6 in organoids at day 60 of culture. **C–F** Percentages of KI67 (C), pH3S10 (D), PAX6 (E), TUNEL (F) positive cells among DAPI^+^ cells in organoids at day 60 of culture. *n* = 3 independent experiments. **G** Ratio of TUJ1^+^ layer to the whole thickness of cortex-like structure in organoids after 60 days of organoids culture. **H** Representative traces of mEPSC recordings in neurons of MECP2-KO and PX330 organoids at day 120 of culture. Scale bars, 5 pA and 1 s. **I** Quantification of mEPSC frequency in PX330 and MECP2-KO organoids. *n* = 11–19 neurons from 4 organoids per group. **J** Quantification of mean mEPSC amplitude in PX330 and MECP2-KO organoids. *n* = 11–19 neurons from 4 organoids per group. *p < 0.05, **p < 0.01, ***p < 0.001. Data are presented as mean ± SEM; Two-tailed Student’s *t-*test

To investigate whether MECP2 was essential in human cortical organoids, we adopted a three-dimensional (3D) culture system to generate cortical organoids from hESCs that closely recapitulated aspects of human fetal neurodevelopment [2, 17, 18]. We generated cortical organoids through sequential changes of media to promote neural induction and neuronal maturation (Fig. 2A). The cell-cycle marker KI67 was mostly located in the radial glial (RG) cell layer, with cells undergoing mitosis at the apical edge, whereas β-III-Tubulin (TUJ1) positive young neurons were located on the basal side of this layer (Additional file 1: Fig. S5A). Next, we determined the effects of MECP2 on hNPs proliferation and differentiation in organoids at day 30. The percentage of cells positive for KI67 and the mitosis-specific cell-cycle marker pH3S10, as well as PAX6, a typical marker for cortical hNPs, among DAPI^+^ cells were not significantly changed in MECP2-KO organoids compared to WT (Additional file 1: Fig. S5B–D). In addition, we did not observe any significant changes in the number of TUNEL^+^ cells (Additional file 1: Fig. S5E) and in the band of TUJ1^+^ neurons (Additional file 1: Fig. S5F), suggesting that there were no obvious phenotypes of neurogenesis and cell death in MECP2 KO cortical organoids at day 30 of culture.

As RTT is a progressive neurodevelopmental disorder, we then examined organoids at D60. By quantifying KI67-labeled cycling oRGs, we found a decrease in cycling oRGs in MECP2 mutant organoids (Fig. 2B, C). Western blotting assay was then validated that the protein level of the proliferation marker PCNA was dramatically reduced in MECP2-mutant organoids at day 60 of culture (Additional file 1: Fig. S5G, H; Additional file 2: D). The percentage of cells positive for pH3S10 as well as PAX6 among DAPI^+^ cells markedly decreased in MECP2 KO organoids (Fig. 2D, E). These results suggested a hNPs reduction in VZ-like regions of MECP2 KO organoids, which was consistent with the results we obtained in MECP2-KO hNPs (Additional file 1: Fig. S4B). Meanwhile, we found a significant reduction of TUJ1^+^ neurons and a significant increase of TUNEL^+^ cells in MECP2-KO organoids (Fig. 2F, G). Together, these results suggested that loss of MECP2 impaired neurogenesis in human cortical organoids.

To further test the maturation defects of MECP2-KO hESC-derived organoids, we performed electrophysiological recordings of neurons in organoids using whole-cell patch recordings at day 120. We found that, in the PX330 group, 44.4% of the recorded mature action potential (AP) firing properties with faster and consistent AP velocity, and 50% fire single or fewer spikes of APs, 5.6% of the recorded cells failed to show spikes of APs (Additional file 1: Fig. S6A). As expected, the proportion of immature neurons and no-spike cells increased in MECP2-KO organoids, indicating that MECP2 deletion affects the maturity of neurons in organoids (Additional file 1: Fig. S6B). And sodium/potassium current exhibited downward trends in MECP2-KO organoids compared to PX330 (Additional file 1: Fig. S6C–E), indicating that MECP2 deletion have impaired electrophysiological features. Next, we performed miniature excitatory postsynaptic currents (mEPSCs) recordings as a way of measuring intercellular connectivity and network formation. At day 120 of culture, the mean frequency and amplitude of mEPSCs were markedly reduced in MECP2-KO organoids compared with PX330 (Fig. 2H–J), indicating reduction in the excitatory synapses. Presumably, this is due to the decreased dendrite length and neuronal complexity observed in MECP2 knockdown neurons (Fig. 1E–H). Collectively, the electrophysiological data supported the changes of synaptic activity in brain organoids after the deletion of *MECP2*.

### Global transcriptional changes in MECP2-KO cortical organoids

**Fig. 3:**
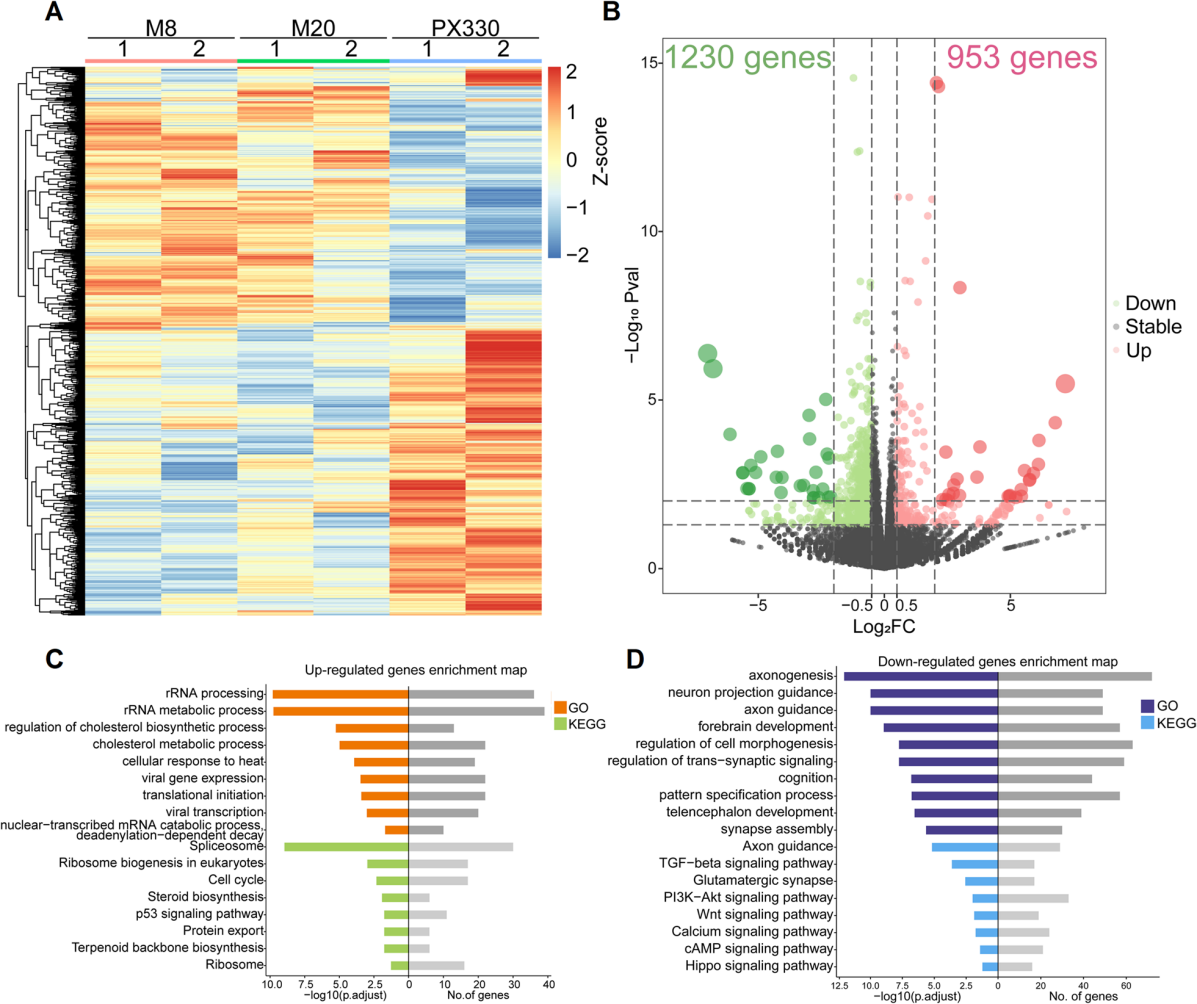
Global transcriptional changes in hESCs-derived MECP2-KO organoids. **A** Heatmap and hierarchical clustering analysis of the significant genes from RNA-seq data. Samples of control (PX330), M8 and M20 were compared. The yellow indicates increased expression of genes, whereas blue stands for the decreased genes compared to PX330. **B** The volcano plots depicting the gene expression changes for M8 or M20 versus PX330. The upregulated, unchanged and downregulated genes are highlighted in red, gray and green, respectively. **C** Bar plots showing the top 9 significantly enriched GO terms (biological processes, BP) and the top 8 significantly enriched KEGG pathways of upregulated genes for M8 and M20 versus PX330. The numbers in the right indicate the number of genes with indicated expression changes. **D** Bar plots showing the top 10 significantly enriched GO terms (biological processes, BP) and the top 8 significantly enriched KEGG pathways of downregulated genes for M8 and M20 versus PX330. The numbers in the right indicate the number of genes with indicated expression changes

To investigate the molecular basis underlying MECP2-regulated neurogenesis, we carried out RNA-seq in MECP2-KO and WT cortical organoids. Unbiased cluster heatmap showed that MECP2 deletion dramatically altered the transcriptome of cortical organoids (Fig. 3A). Compared to the WT, we found that 953 and 1230 genes were identified upregulated and downregulated for at least 1.5 folds in MECP2 KO organoids, respectively (*p* < 0.05) (Fig. 3B). Strikingly, GO (Gene Ontology) term and KEGG (Kyoto Encyclopedia of Genes and Genomes) pathway analysis showed that genes upregulated in MECP2 KO organoids were enriched in rRNA processing, p53 signaling pathway (Fig. 3C), suggesting that the effect of cell death in MECP2 KO cortical organoids may be due to the induction of p53 pathway. While downregulated genes were involved in PI3K-AKT, TGF-β, Wnt or Hippo signaling pathways which were important for axonogenesis, neuron projection guidance, axon guidance, regulation of trans-synaptic signaling and forebrain development (Fig. 3D). This is in agreement with our observations showing that neurons in MECP2-KO organoids displayed a decreased neuronal maturity caused mainly by alterations in excitatory neurotransmission. The global changes in the expression of neurodevelopment-related gene might cause neuronal deficits in MECP2-KO cortical organoids. Moreover, the transcriptome analysis suggested that this disease-in-a-dish organoid model can provide relevant insights into RTT.

### Treatment with KW-2449 or VPA alleviates the morphological deficits in MECP2-KO neurons

**Fig. 4:**
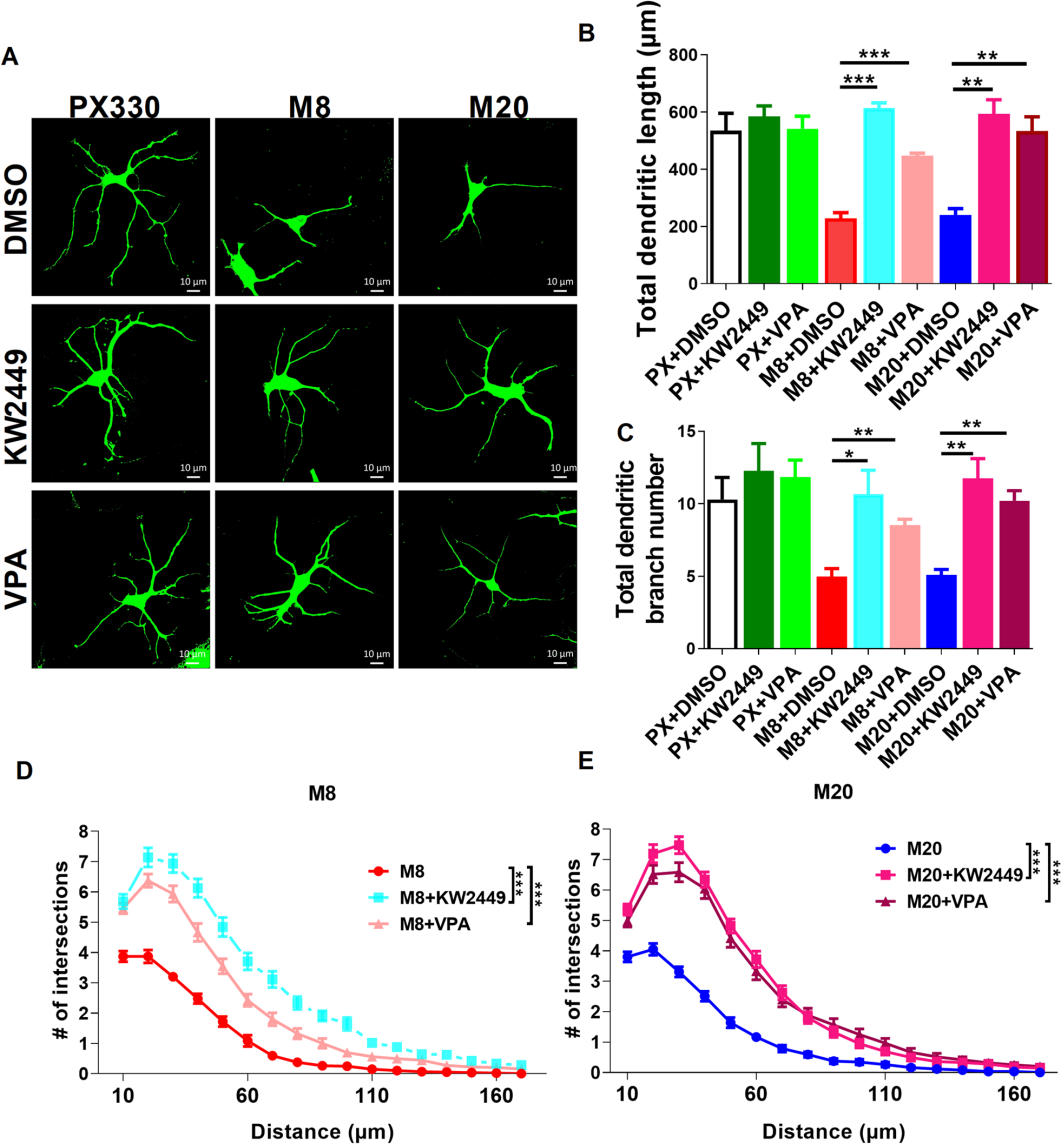
KW-2449 and VPA restores human MECP2-KO neurons in 2D culture. **A** Representative image of immunofluorescence staining for MAP2 in PX330 and MECP2-KO neurons treated with 1 µM KW-2449 or 10 µM VPA during days 20–40 of neural differentiation. **B–E** Quantification of total dendritic length (**B**), dendritic branch number (**C**), and intersection numbers (**D**, **E**) of MAP2^+^ neurons in **A**. *n* > 60 neurons from 3 independent experiments per group. *p < 0.05, **p < 0.01, ***p < 0.001. Data are shown as mean ± SEM; Two-tailed Student’s *t*-test

As we found global dysregulation of transcription in MECP2 KO organoids, we sought to identify small molecules that are capable of broadly altering transcription rather than affecting a single gene target. Our RNA-seq analysis showed that many PI3K-AKT pathway-related genes (e.g. *PIK3R1*, *ITGA8*, *LAMC3*, *SYK, MAGI1*, *EGF*, *TNR*, *FLT1*, *VTN*, *FGF9*, *KIT*, *ITGB8*, *ITGA9*, *SGK3*, *TNC*, *THBS4*, *GNG4*, *LAMA2*, *LAMB1*) were downregulated in MECP2-KO cortical organoids. Since PI3K-AKT pathway-related genes play crucial roles in dendrite structure and synaptic plasticity, which are often impaired in RTT patients [19], we then speculated that activation of PI3K-AKT pathway might rescue neuronal deficits in MECP2-KO cortical organoids. KW-2449 has been applied as a FLT3 inhibitor in clinical trials [20], and KW-2449 may activate activity-dependent genes such as BDNF and Akt-mTOR pathway-related genes which regulate cell growth and survival [9, 21]. VPA is an FDA-approved antiepileptic drug with efficacy against multiple seizure types [22]. VPA activates the PI3K-Akt pathway and reduces neurological symptoms RTT mice [23–25]. Therefore, we tested whether KW-2449 or VPA treatment would alleviate neuronal deficits in MECP2-KO human neuronal models. Indeed, significant enhancement in dendritic length, branch number and morphological complexity was observed in KW-2449 or VPA-treated MECP2-KO neurons (Fig. 4A–E), indicating that the significant improvement during continuous KW-2449 or VPA treatment may be required to achieve functional rescue in MECP2-KO neurons.

### KW-2449 and VPA effectively prevent developmental defects in MECP2-KO brain organoids

**Fig. 5:**
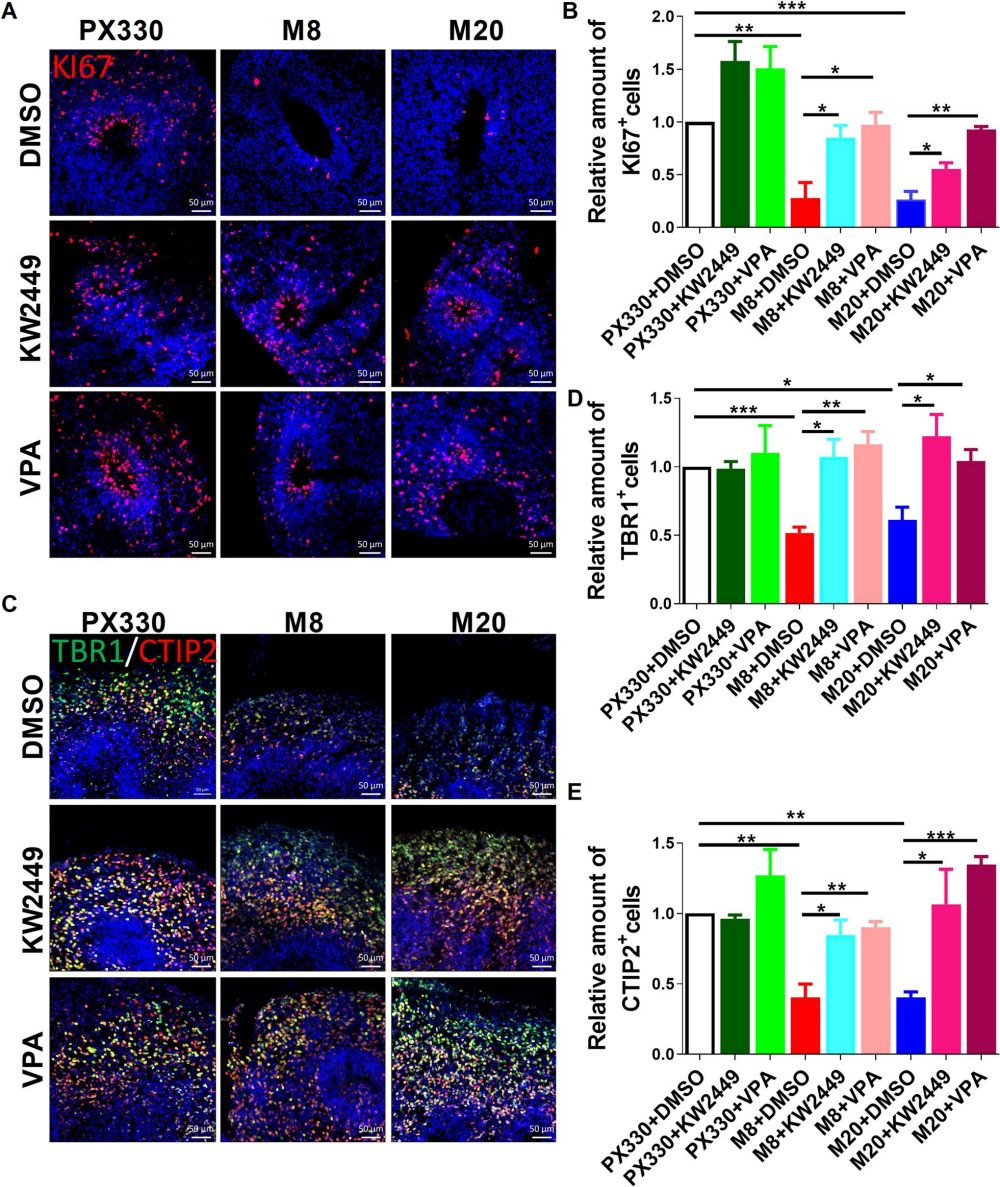
KW-2449 and VPA rescue neurogenesis defects in MECP2-KO organoids. **A**, **B** Representative images (A) and quantitation (B) of immunofluorescence staining for KI67 at day 60 in PX330 and MECP2-KO organoids treated with DMSO (control), 1 µM KW2449 or 10 µM VPA for 49 days. *n* = 3 independent experiments. **C–E** Representative images (**C**) and quantitation (**D**, **E**) of immunofluorescence staining for TBR1 and CTIP2 at day 60 in PX330 and MECP2-KO organoids treated with DMSO (control), 1 µM KW2449 or 10 µM VPA for 49 days. *n* = 3 independent experiments. *p < 0.05, **p < 0.01, ***p < 0.001. Data are shown as mean ± SEM; Two-tailed Student’s *t*-test

To further validate the beneficial effect of KW-2449 or VPA on MECP2-KO neurons, we treated MECP2-KO cortical organoids with KW-2449 or VPA at day 10, and investigated their development at day 60 of culture. As we expected, KI67 staining analysis revealed that KW-2449 or VPA treatment rescued the reduced proportion of cycling hNPs in MECP2-KO cortical organoids at day 60, compared to the DMSO-treated group (Fig. 5A, B). Meanwhile, the relative amounts of TBR1^+^ and CTIP2^+^ post mitotic neurons were significantly increased in KW-2449- or VPA-treated MECP2-KO organoids, compared to those of DMSO-treated group (Fig. 5C-E). These results proved that KW-2449 and VPA had beneficial effect on the development of MECP2-KO cortical organoids.

### KW-2449 or VPA reverses the transcriptome of MECP2-KO cortical organoids

**Fig. 6:**
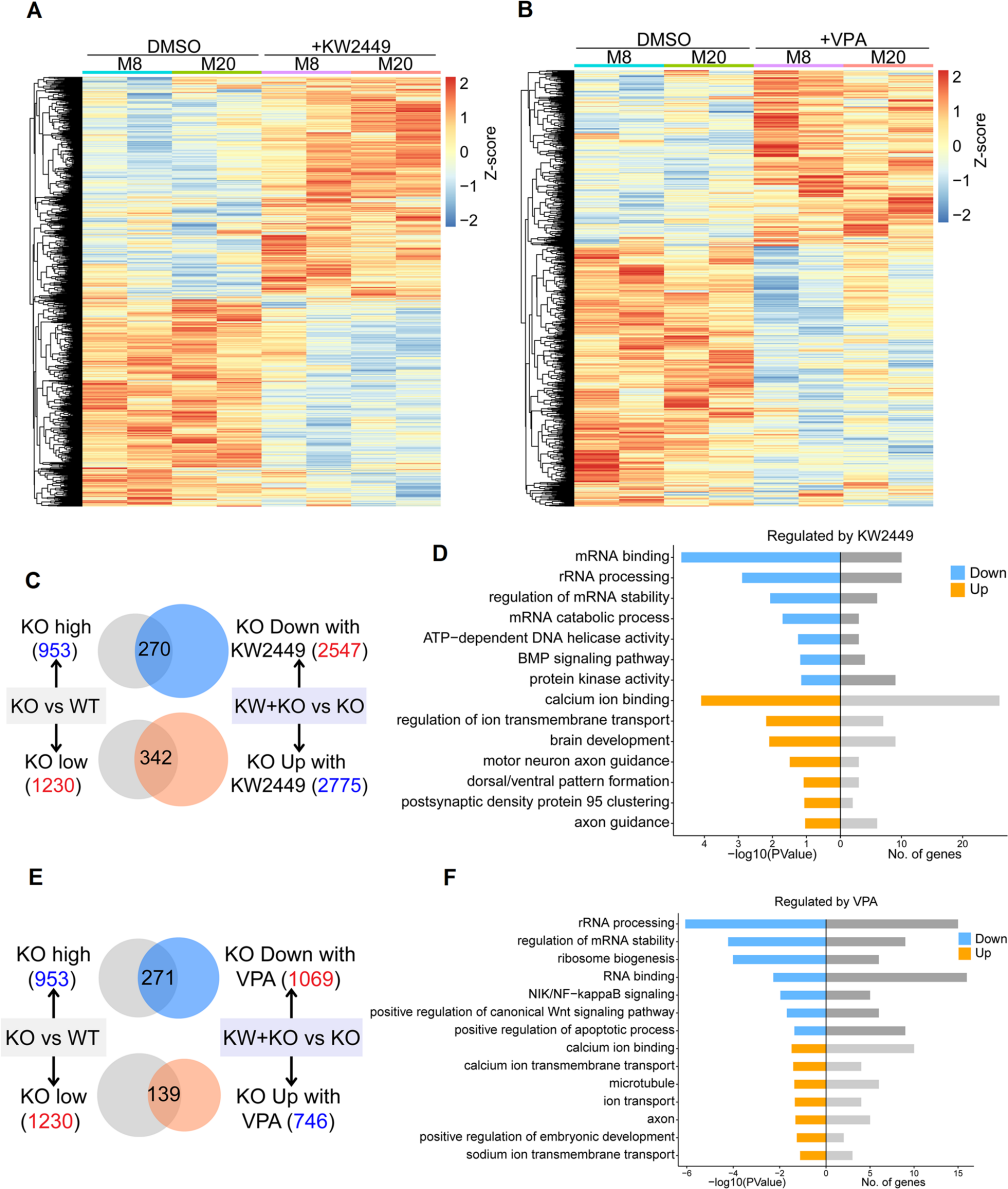
KW2449 and VPA reverse transcriptome changes in MECP2-KO organoids. **A** Heatmap and hierarchical clustering analysis of the significant genes from RNA-seq data. M8 and M20 organoids treated either with DMSO or KW2449 were compared. The yellow indicates increased expression of genes, whereas blue stands for the decreased genes. **B** Heatmap and hierarchical clustering analysis of the significant genes from RNA-seq data. Organoid samples treated with either DMSO or VPA were compared. The yellow indicates increased expression of genes, whereas blue stands for the decreased genes. **C** Venn diagrams of genes affected by KW2449 treatment in MECP2-KO organoids. In MECP2 KO organoids, genes upregulated in comparison with WT were significantly downregulated by KW2449; vice versa, in MECP2 KO organoids, downregulated genes were significantly upregulated by KW2449. **D** Bar plots depicting the top 7 significantly enriched GO terms of dysregulated genes to stimulation response were rescued by KW2449 in MECP2 KO organoids. Upper panel, downregulated genes; Lower panel, upregulated genes. The numbers in the right indicate the number of genes with indicated expression changes. **E** Venn diagrams of genes affected by VPA treatment in MECP2-KO organoids. In MECP2 KO organoids, genes upregulated in comparison with WT were significantly downregulated by VPA; vice versa, in *MECP2* KO organoids, downregulated genes were significantly upregulated by VPA. **F** Bar plots depicting the top 7 significantly enriched GO terms of dysregulated genes to stimulation response were rescued by VPA in MECP2 KO organoids. Upper panel, downregulated genes; Lower panel, upregulated genes. The numbers in the right indicate the number of genes with indicated expression changes

To better understand the underlying molecular mechanisms of beneficial effects from KW-2449 and VPA, we profiled the transcriptomes of MECP2-KO cortical organoids treated with KW2449 or VPA. We found that the transcriptomes of MECP2 KO organoids shifted following KW2449 or VPA treatment (Fig. 6A, B). Specifically, the expression level of 270 upregulated genes in MECP2 KO organoids was dramatically decreased upon KW-2449 treatment, and these 270 genes were associated with protein kinase activity, rRNA processing and BMP signaling pathway (Fig. 6C, D). In contrast, those 342 downregulated genes in MECP2 KO organoids were upregulated upon KW-2449 treatment, and those 342 genes were involved in regulation of calcium ion binding, brain development and axon guidance (Fig. 6C, D).

Similarly, the expression levels of 271 upregulated genes and 139 downregulated genes in MECP2-KO brain organoids were significantly reversed following VPA treatment (Fig. 6E, F). Further GO term analysis demonstrated that these expression-reversed genes were enriched in rRNA processing, Wnt signaling pathways, positive regulation of apoptotic process, calcium ion binding, and axon (Fig. 6F). As we expected, PI3K-Akt pathway-related genes (> 1.5 folds, *p* value < 0.05) were reversed their expressions in MECP2-KO organoids after KW2449 treatment. These genes included the followings: *GHR*, *CDKN1A*, *COL4A5*, *ITGA3*, *PRLR*, *IGF1*, *MCL1*, *CDK6*, *COL4A6*, *LAMA5*, *BCL2L1*, *CCND1*, *GYS1*, *COL9A2*, *FGFR2*, *CSF1*, *SGK1*, *NTF3*, *NTRK2*, *COL6A1*, *MYC*, *CREB3L2*, *IRS1*, *THEM4*, *LAMC1*, *FGF2*, *ANGPT2*, *THBS3*, *LAMB1*, *RPS6*, *DDIT4*, *ITGB4*, *MET*, *SGK3*, *ITGA7*, *COL2A1*, *HSP90B1*, *CSF1R*, *VEGFA*, *PCK2*, *COL6A2*, *ERBB2*, *ITGB5*, *EPHA2*, *ATF6B*, *PPP2R3B*, *PDGFC*, *RELN*, *PPP2R2B*, *IL2RG*, *GNG5*, *GNG12*, *LAMA3*, *COL9A1*, *VEGFB*, *G6PC3*, *PPP2R5A*, *HRAS*, *ATF4*, *BRCA1*, *BAD*, *LAMC2*, *GNG10*, *AKT2*, *BDNF*, *TNC*, *ITGA11*, and *YWHAE*. Moreover, KEGG analysis indicated that these up-regulated genes (> 1.5 folds, *p* value < 0.05) were involved in PI3K-Akt signaling pathway in MECP2-KO organoids treated with KW2449. Consistently, there were 19 genes (*PIK3R1*, *ITGA8*, *LAMC3*, *SYK, MAGL1*, *EGF*, *TNR*, *FLT1*, *VTN*, *FGF9*, *KIT*, *ITGB8*, *ITGA9*, *SGK3*, *TNC*, *THBS4*, *GNG4*, *LAMA 2*, *LAMB1*) that were associated with PI3K-Akt signaling pathway (downregulated in MECP2-KO group in Fig. 3D) were shifted their expression from downregulation to upregulation in MECP2-KO organoids after the treatment of VPA.

Taken together, these results supported the idea that there was a transcriptomic switch in MECP2-KO cortical organoids after the treatment of either KW-2449 or VPA.

## Discussion

Three-dimensional organoids provide unique platforms for modeling human brain development and neurological disease in an experimentally tractable and abundant form [18, 26, 27]. In this study, we have combined hESCs and CRISPR-Cas9 gene editing technology to develop a potential drug screening platform for RTT. This study provides new evidence showing that MECP2 depletion caused the deficits in dendritic morphology when we differentiated hESCs into 2D neurons and reduced cortical progenitor cells and neurons in 3D brain organoids. Further, we found the global transcriptome changes between WT and MECP2-KO organoids. GO analysis of DEGs showed that downregulated genes were enriched in the categories of neuronal functions of axonogenesis, neuron projection guidance, axon guidance, forebrain development and regulation of trans-synaptic signaling. Our KEGG analysis indicated that MECP2 knockout was related to the dysfunction of PI3K-AKT, TGF-β, Wnt, Hippo signaling and cAMP signaling pathway.

Actually, dysregulation of the AKT/mTOR axis has been observed in MeCP2-null mice as well as in human RTT neurons [19, 21, 28]. MECP2 regulates early human neurogenesis through differential effects on ERK and AKT signaling [29]. Of note, BDNF, a biomarker for RTT, is well-known for activating PI3K/Akt and for the regulation of neuronal function [19, 28, 30–32]. The present study identifies BDNF and dozens of PI3K-AKT pathway-related genes were significantly downregulated in MECP2-KO cortical organoids and provides strong evidence that activation of PI3K-AKT pathway efficiently rescues neuronal deficits in MECP2-KO cortical organoids.

In the present study, we observed that MECP2 deletion caused simplified dendritic morphology and impaired synaptic activity which were similar to a few publications studying RTT using various RTT models. Marchetto and colleagues showed that neurons derived from RTT-iPSCs had fewer synapses, reduced spine density and electrophysiological defects studies [29, 33–37]. Studies in murine models of MeCP2 deficiency implicate an accordant deficit in synaptic function, including decreased synaptic transmission and plasticity [38–40). Moreover, electrophysiological phenotype of altered synaptic physiology was also reported in several other RTT models [41–44]. At the molecular level, a spectrometry-based quantitative proteomic analysis of neural differentiation of RTT hiPSCs revealed that downregulated genes were involved in synaptogenesis, dendritic morphology, excitatory postsynaptic potential and forebrain development pathways, while upregulated genes were involved in neuron apoptotic process, cell–cell adhesion, rRNA processing, acyl-CoA metabolic process, translational initiation, and DNA repair-related pathways [45]. Similarly, our RNA-seq analysis also supported that dysregulated genes in MECP2-KO cortical organoids were enriched in neuron projection guidance, axon guidance, forebrain development and regulation of trans-synaptic signaling, rRNA processing, translational initiation, and P53 signaling pathway. Although mutation of MECP2 alters the expression of thousands of genes, the effects of an individual gene are usually small, indicating MECP2 acts as a global regulator of gene expression and chromatin architecture that mediates cellular changes through mediation of a great number of genes genome-wide [46]. The present study supports that mutation of MECP2 causes a genome-wide alteration of gene expression during brain organoid development. Gene candidates that we presented in this study could be worthy to explore the roles of MECP2 and to develop therapeutic intervention for RTT patients.

Previous efforts to identify therapeutic targets have mostly focused on testing in 2D neurons such as IGF1, HDAC6 inhibitor and KCC2 [47]. To overcome the limitations of 2D cultures, we established 3D organoids from MECP2-KO hESCs and identified two small-molecule compounds KW-2449 and VPA that restored the morphological abnormalities. Also, consistent with the transcriptomic analysis, the expression levels of calcium ion transport and axon-related genes recovered after KW-2449 or VPA treatment of MECP2-KO organoids. As shown by GO analysis, transcription of genes involved in PSD95 clustering and brain development were improved in MECP2-KO organoids by KW2449 treatment and genes involved in microtubule and positive regulation of embryonic brain development were improved in MECP2-KO organoids by VPA treatment. Our results indicate that KW2449 or VPA can rescue brain organoids deficits caused by MECP2 loss-of-function.

In consistent with previous findings that injection of KW-2449 ameliorates disease-associated respiratory and locomotion phenotypes in MeCP2 mutant mice [48], we provide important clues for KW2449 in further clinical research for RTT treatment in the future. Given that phase I clinical trials may provide a more sensitive assay for a candidate drug’s toxicity and safety compared with conventional clinical trial phases [10], the integrative approach of mimicking RTT using patient-derived hiPSC and gene-edited hESCs are critical for drug screening and for therapeutic applications in future studies.

## Conclusion

Taken together, our data provide insights into the three-dimensional organoids of RTT for exhibiting abnormal phenotypes and for testing potential drugs. We find the efficacy of KW2449 or VPA in rescuing a number of well-documented deficits in RTT. Although our current research has revealed the transcriptome dysregulation in brain organoid after MECP2 deletion, further functional experiments are required to examine those potential downstream targets of MECP2 in brain organoids that are differentiated from both patient-derived hiPSC and gene-edited hESCs.

## Supplementary Information

**Additional file 1**. Supplemental figures and figure legends.

**Additional file 2**. WB original pictures.

**Additional file 3**. Examining the potential off-target sites in hESCs.

## Data Availability

The RNA-sequencing data have been deposited in the NCBI GEO database (www.ncbi.nlm.nih.gov/geo/) and are available under the accession number GSE214885. The data that support the findings of this study are available from the corresponding author upon reasonable request.

## Abbreviations

RTT: Rett syndrome
ESCs: Human embryonic stem cells
MECP2: Methyl-CpG binding protein 2
HDAC: Histone deacetylase
KO: Knock-out
mEPSCs: Miniature excitatory postsynaptic currents
AP: Action potential
EBs: Embryoid bodies
