# Supplementary material for: KW-2449 and VPA exert therapeutic effects on human neurons and cerebral organoids derived from MECP2-null hESCs
Source: Stem Cell Res Ther. 2022 Dec 27;13:534. doi: 10.1186/s13287-022-03216-0 (PMC9795779; doi:10.1186/s13287-022-03216-0)
Supplement: Supplementary file 1 — Additional file 1. Supplemental figures and figure legends. [file 13287_2022_3216_MOESM1_ESM.docx]

**Supplemental Figures and Figure Legends**


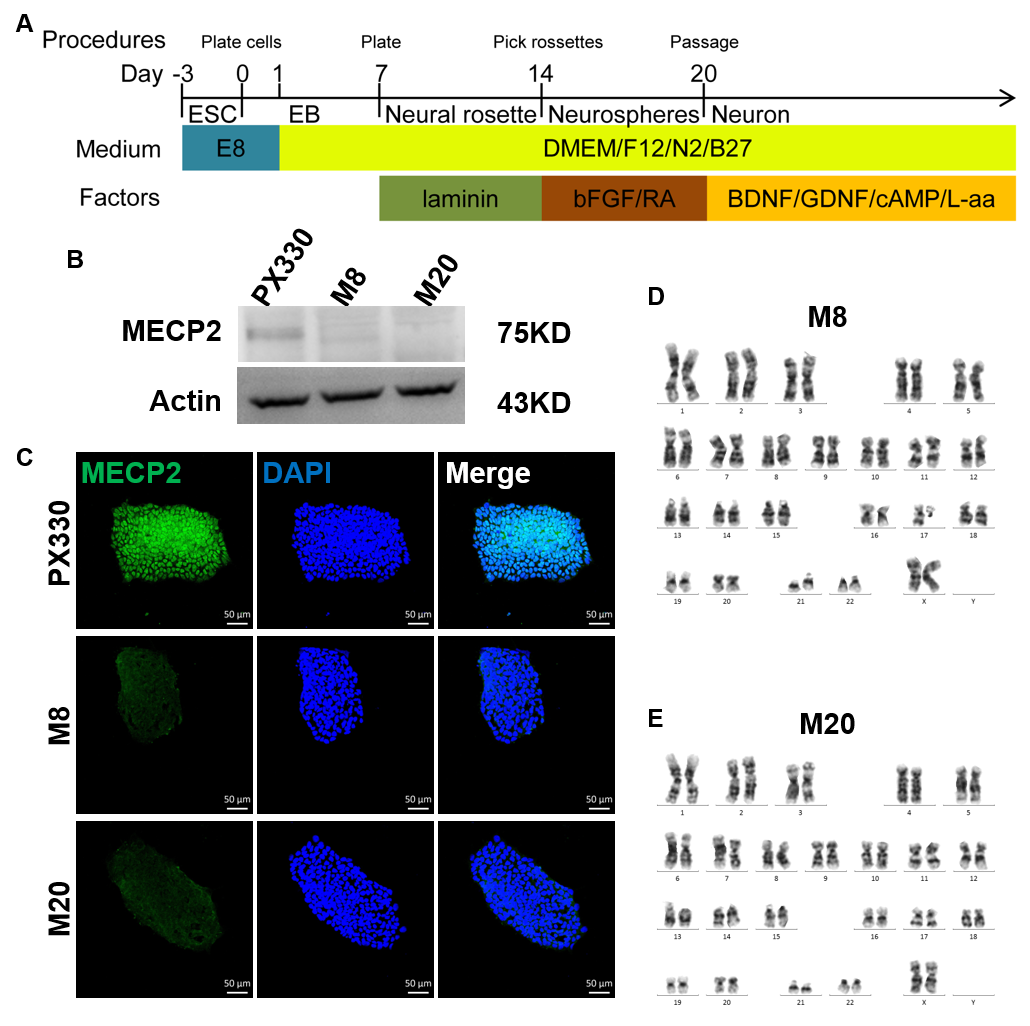


### Fig. S1 *MECP2* deletion doesn’t induce abnormal karyotype in hESCs

**A** Schematic diagram of the strategy to differentiate neurons from hESCs. **B** Western blotting analysis depicting MECP2 protein is undetectable in M8 and M20 clones compared to PX330. **C** Representative images of MECP2 immunofluorescence staining in hESCs showing MECP2 signal is nearly undetectable in MECP2-KO clones compared to PX330. **D, E** Karyotype analysis for MECP2 KO hESCs. Upper panel, karyotype of M8; Lower panel, karyotype of M20.


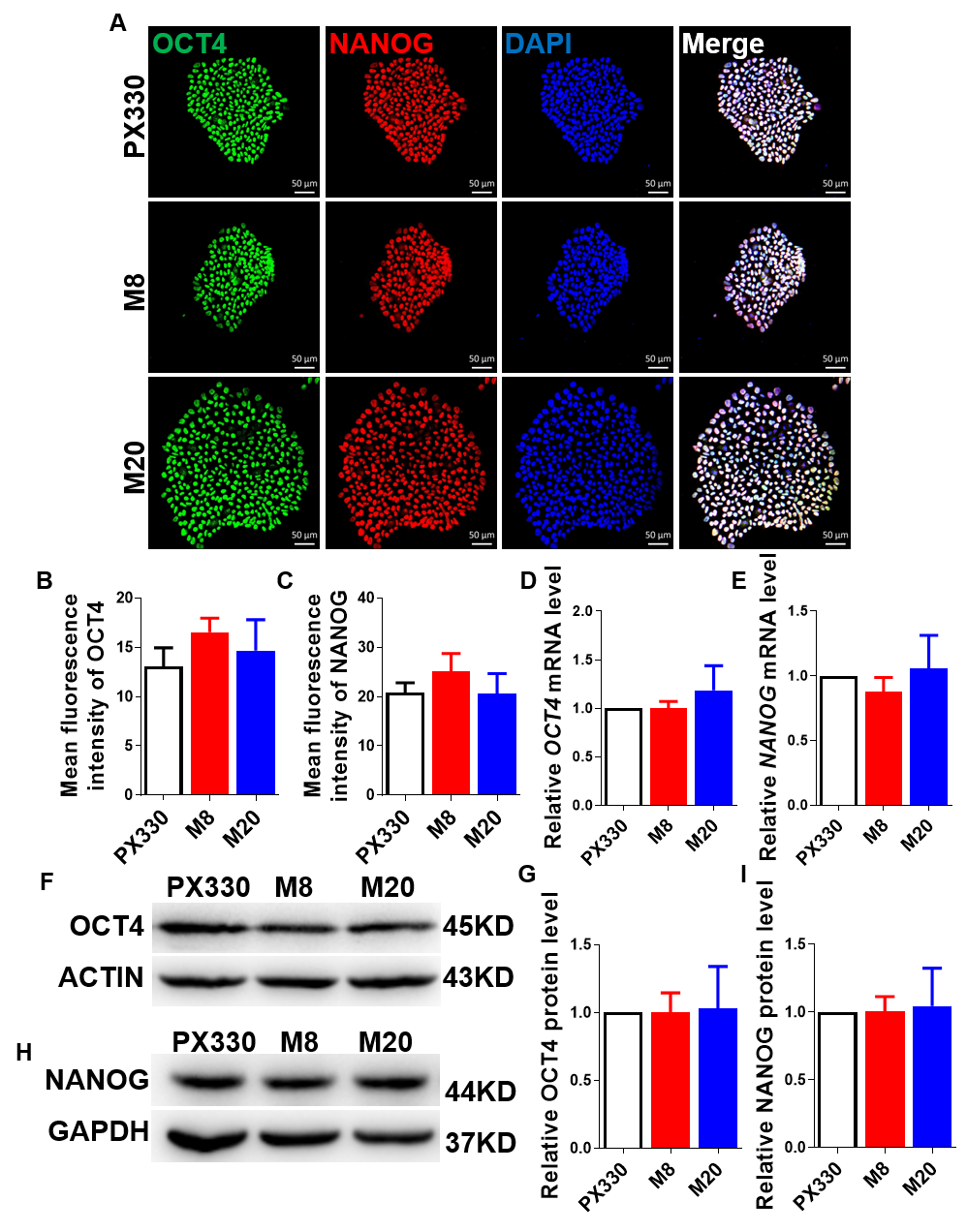


### Fig. S2 The deletion of *MECP2* doesn’t change the pluripotency of hESCs

**A-C** Representative images (A) and quantification (B, C) of immunofluorescence staining for pluripotency markers OCT4 and NANOG in WT and MECP2-KO hESCs. n = 3 independent experiments. **D, E** Validation of *OCT4* and *NANOG* mRNA levels in WT and MECP2-KO hESCs by qRT-PCR. The expression levels were normalized to *GAPDH*. n = 3 independent experiments. **F-I** OCT4 and NANOG protein expressions in WT and MECP2 KO hESCs by western blot. n > 3 independent experiments. Data are presented as mean ± SEM; Two-tailed Student’s *t*-test.


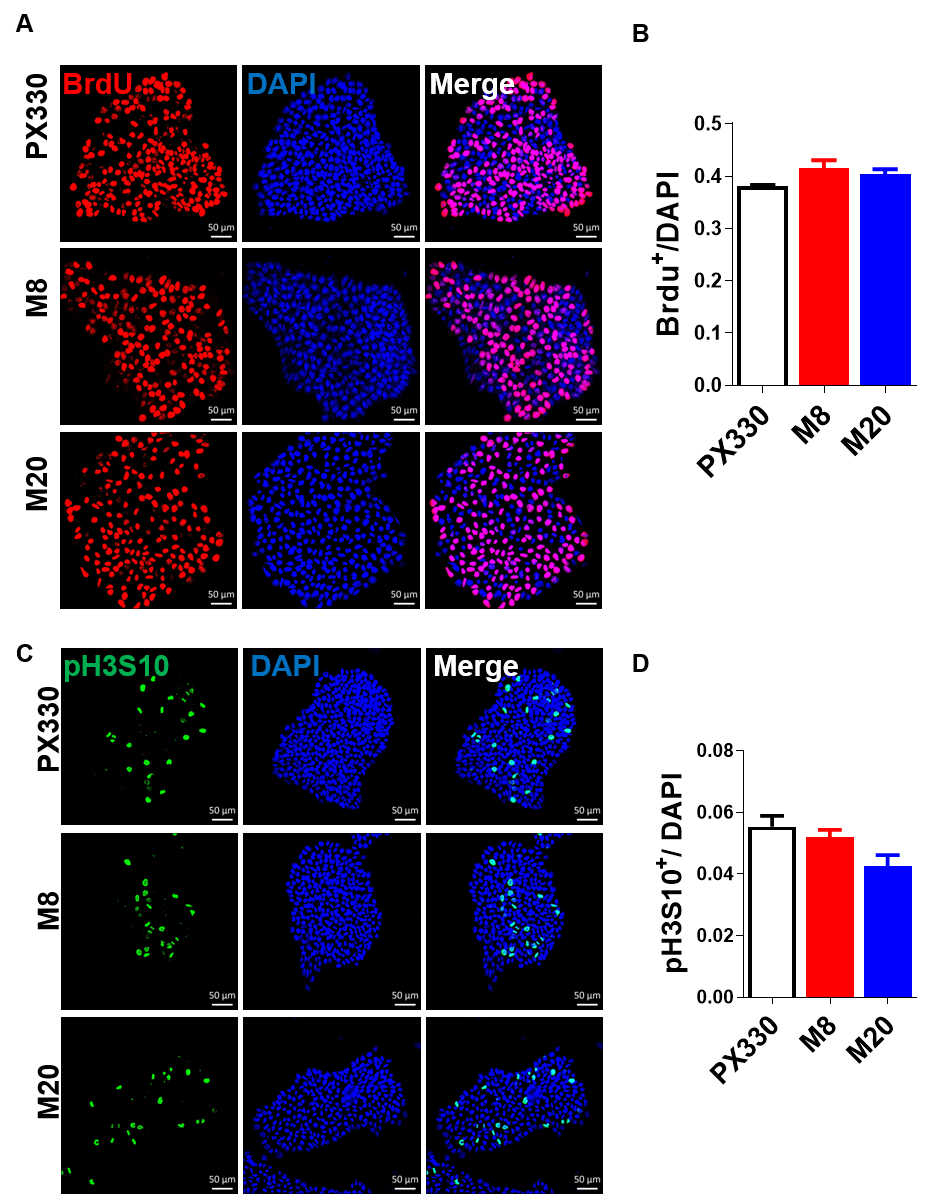


### Fig. S3 The deletion of *MECP2* doesn’t change the self-renewal of hESCs

**A, B** Representative images (A) and quantification (B) of immunofluorescence staining for BrdU in WT and MECP2-KO hESCs. n = 3 independent experiments. **C, D** Representative images (C) and quantification (D) of immunofluorescence staining for pH3S10 in WT and MECP2-KO hESCs. n = 3 independent experiments. Data are presented as mean ± SEM; Two-tailed Student’s *t*-test.


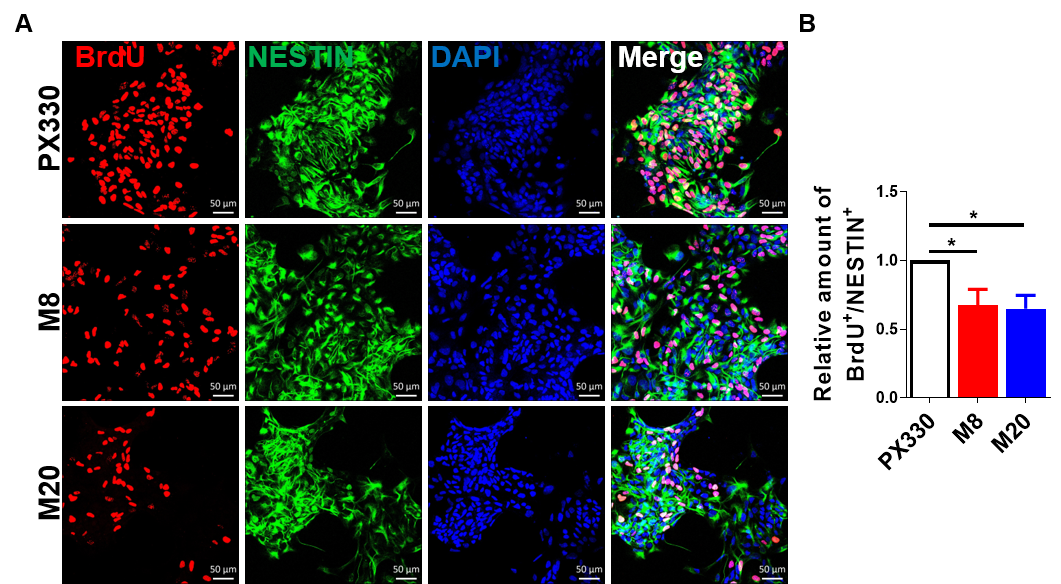


### Fig. S4 Lacking of MECP2 impairs neural stem cell proliferation

**A, B** Representative images (A) and quantification (B) of immunofluorescence staining for BrdU and NESTIN in WT and MECP2-KO hNPs. n = 3 independent experiments. **p* < 0.05. Data are shown as mean ± SEM; Two-tailed Student’s *t*-test.


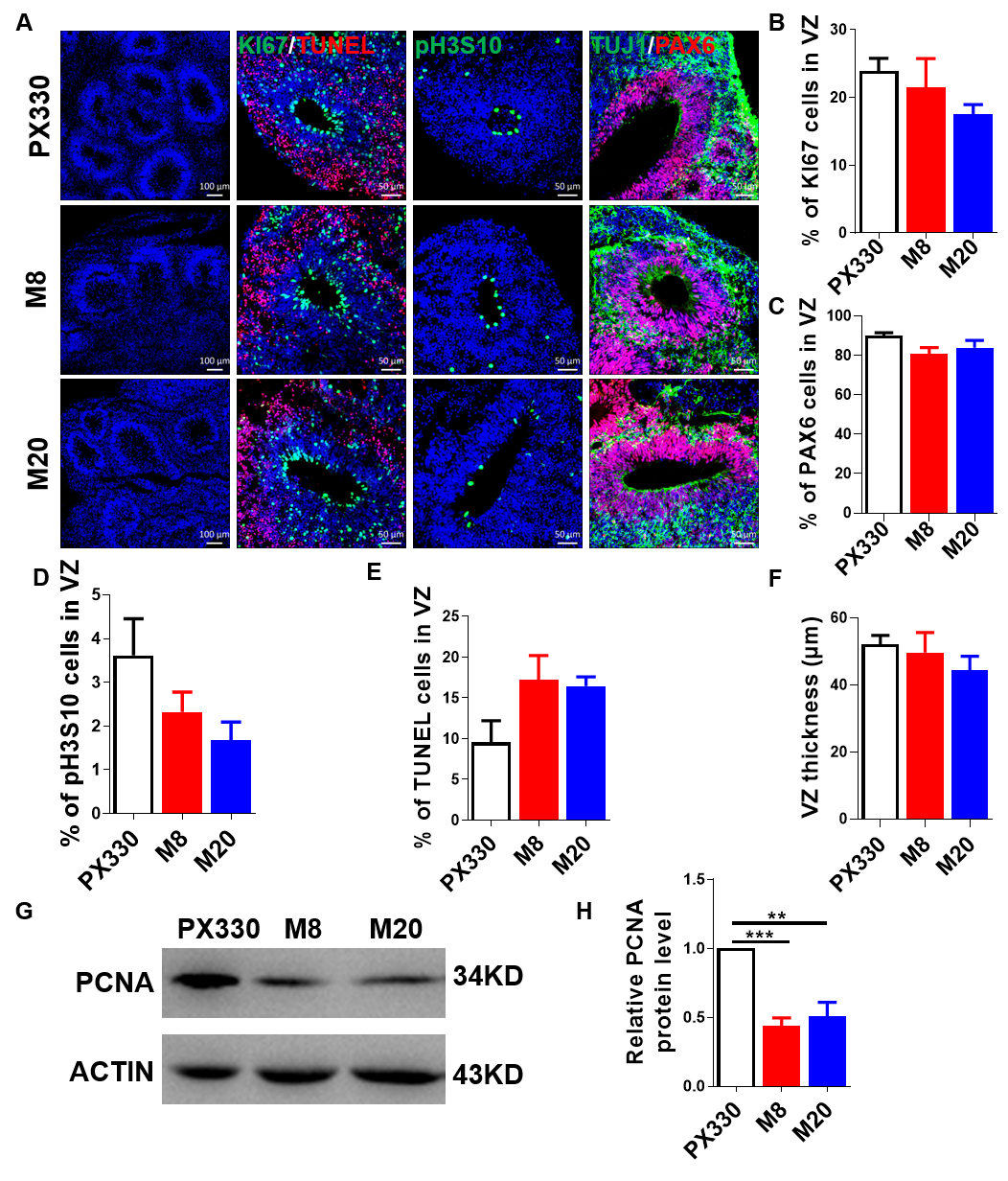


**Fig. S5 Regulation of organoid development by MECP2.**

**A** Representative image of immunofluorescence staining for KI67, TUNEL, pH3S10, TUJ1 and PAX6 in organoids at day 30 of culture. **B-F** Percentages of KI67^+^ (B), PAX6^+^ (C), pH3S10^+^ (D), TUNEL^+^ (E) cells among DAPI^+^ cells, and the band of TUJ1 thickness (F) in organoids at day 30 of culture. n = 3 independent experiments. **G, H** Representative images (G) and quantification (H) of western blotting for PCNA in WT and MECP2-KO organoids at day 60 of culture. n = 3 independent experiments. ***p* < 0.01, ****p* < 0.001. Data are shown as mean ± SEM; Two-tailed Student’s *t*-test.


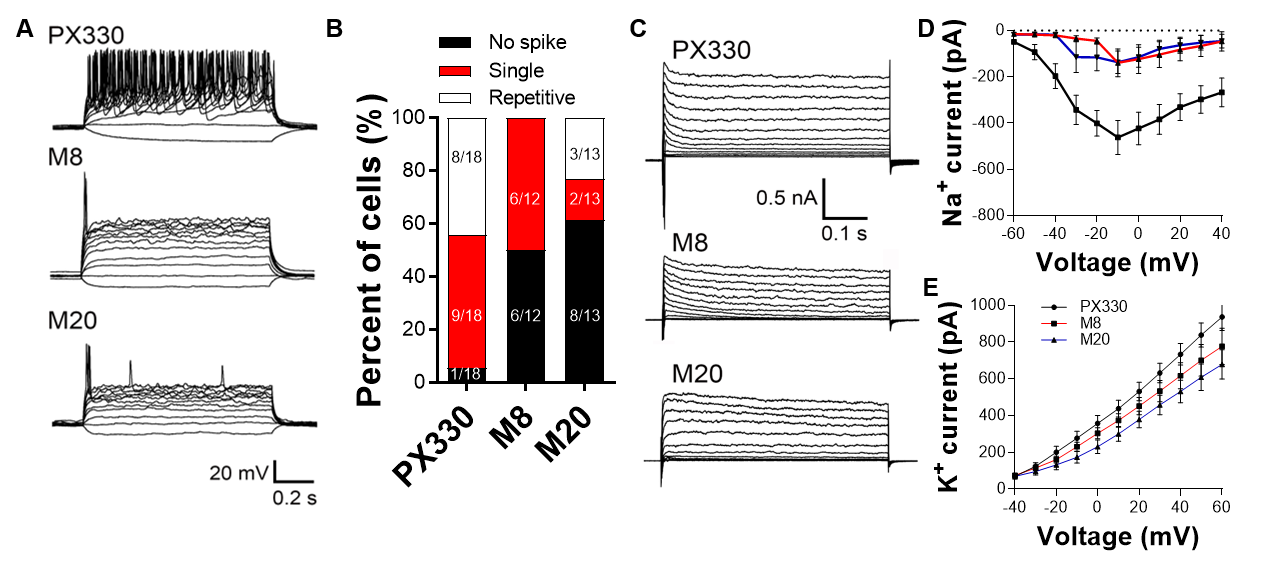
 **Fig. S6 Loss of MECP2 leads to electrophysiological defects in hESCs-derived organoids.**

**A** Representative traces of membrane potential responding in MECP2-KO and PX330 organoids. Depolarization by current injection steps from -5 pA to +50 pA in 5 pA increments. Membrane potential was current-clamped at around -65 mV. **B** Quantification of neuronal maturity by recorded AP firing patterns in organoids at day 120 during differentiation (n > 12 neurons from 3 organoids in each group). **C** Representative traces of whole-cell currents in voltage-clamp mode of MECP2-KO and PX330 organoids. Neurons were held at -60 mV. Step depolarization from -60 to +60 mV at 10 mV intervals was delivered. **D** Averaged current-voltage relationship (I-V curves) for Na^+^ currents in organoids at day 120 of culture. **E** Averaged current-voltage relationship (I-V curves) for K^+^ currents in organoids at day 120 of culture. Data are shown as mean ± SEM.
