# Supplementary material for: KW-2449 and VPA exert therapeutic effects on human neurons and cerebral organoids derived from MECP2-null hESCs
Source: Stem Cell Res Ther. 2022 Dec 27;13:534. doi: 10.1186/s13287-022-03216-0 (PMC9795779; doi:10.1186/s13287-022-03216-0)
Supplement: Supplementary file 2 — Additional file 2. WB original pictures. [file 13287_2022_3216_MOESM2_ESM.docx]

**Supplemental Figures and Figure Legends**


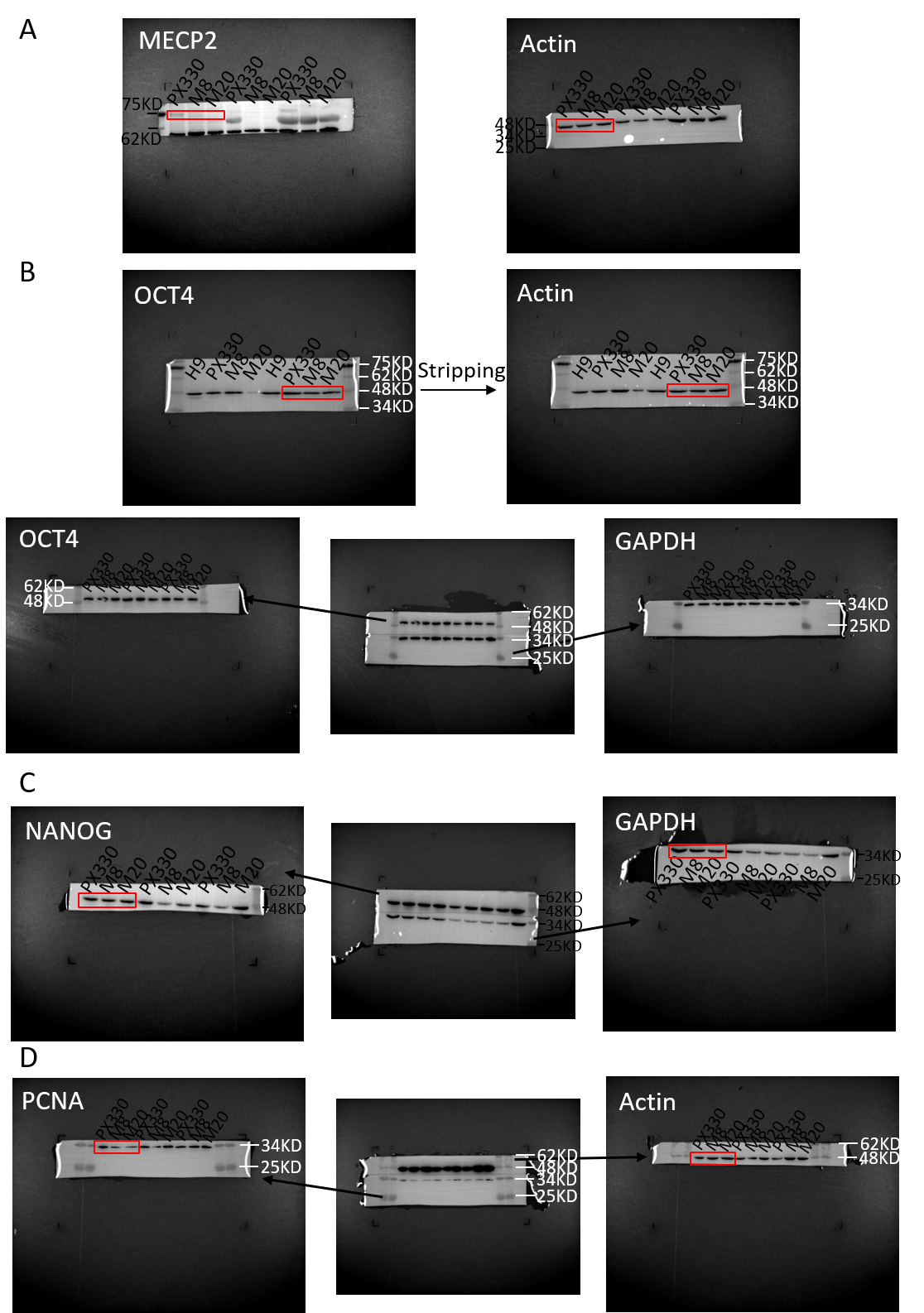


Additional file 2: WB original pictures. **A** WB original pictures in Fig. S1B. The representative images in Fig. S1B are highlighted in red. **B** WB original pictures in Fig. S2F. The representative images in Fig. S2F are highlighted in red. **C** WB original pictures in Fig. S2H. The representative images in Fig. S2H are highlighted in red. **D** WB original pictures in Fig. S5G. The representative images in Fig. S5G are highlighted in red.
